# Respiratory syncytial virus vaccination in systemic autoimmune rheumatic diseases: a systematic review

**DOI:** 10.1093/rheumatology/keag513

**Published:** 2026-09-17

**Authors:** Victoria Allen, Maryam Adas, Liyang Pan, Nilay Joshi, Mark Russell, Katie Bechman, James Galloway

**Affiliations:** Centre for Rheumatic Diseases, King’s College London, London, UK; Centre for Rheumatic Diseases, King’s College London, London, UK; College of Medicine, University of Jeddah, Jeddah, Saudi Arabia; Queen Elizabeth Hospital, London, UK; Kettering General Hospital NHS Foundation Trust, Kettering, UK; Centre for Rheumatic Diseases, King’s College London, London, UK; Centre for Rheumatic Diseases, King’s College London, London, UK; Centre for Rheumatic Diseases, King’s College London, London, UK

**Keywords:** vaccines, respiratory syncytial virus (RSV), systematic review, immunocompromise, safety

## Abstract

Respiratory syncytial virus (RSV) vaccination is available for older adults. Patients with systemic autoimmune rheumatic diseases (SARDs), including those receiving immunosuppressive therapies, were excluded from trials. Evidence to guide vaccination decisions is limited. We conducted a systematic review evaluating RSV epidemiology, vaccine effectiveness, immunogenicity and safety in adults with SARDs. Databases were searched from inception to 28 January 2026. Observational studies, clinical trials and pharmacovigilance reports were included. Evidence specific to disease-modifying antirheumatic drugs (DMARDs) was examined where available. Of the 249 unique records returned, 43 met the inclusion criteria. Six studies were identified in the supplementary search, yielding 49 studies in total. Only one study reported RSV outcomes in SARD patients. No study evaluated vaccine effectiveness stratified by DMARD class. Observational data suggest autoimmune and immunocompromising conditions are associated with increased RSV-associated hospitalization, although attribution is uncertain due to bias and data limitations. RSV vaccines demonstrated first-season effectiveness of 60–73% in immunocompromised adults, with evidence of attenuated and more rapidly waning protection compared with immunocompetent populations. Immunogenicity studies from RSV and other vaccine platforms indicate reduced seroconversion in immunosuppressed patients. No consistent autoimmune safety signals were identified, though data in SARD populations remain limited. RSV poses a relevant, though incompletely quantified, risk for SARD patients. Vaccination appears effective and safe in immunocompromised populations, but protection may wane more rapidly. The absence of DMARD-specific data represents an evidence gap. Until further evidence emerges, vaccination should be considered for eligible patients using a personalized framework.

*Rheumatology* key messages

- RSV vaccination appears safe and effective in immunocompromised adults, despite limited SARD-specific evidence.
- SARD patients may experience increased RSV hospitalization risk, with faster waning vaccine protection.
- Absence of DMARD-specific evidence highlights need for personalized RSV vaccination decisions in eligible SARD patients.

## Introduction

Respiratory syncytial virus (RSV) causes substantial morbidity and mortality in older adults. with ∼336 000 hospitalizations and 14 000 in-hospital deaths annually among adults aged ≥65 years worldwide. Case fatality rates approach 8% in hospitalized patients [1, 2]. Unlike the paediatric disease burden, adult RSV illness remained under-recognized for decades. Severe acute respiratory syndrome coronavirus 2 (SARS-CoV-2), and its accompanying expansion of multiplex respiratory viral panels, brought renewed attention to RSV as a significant pathogen in adults [3, 4].

The approval of the first RSV vaccines for older adults in 2023 represented a key moment in respiratory virus prevention. Two protein-subunit vaccines—RSVpreF (Abrysvo; Pfizer) and RSVPreF3-AS01 (Arexvy; GSK)—demonstrated efficacy against RSV-associated lower respiratory tract disease in phase 3 trials, with vaccine efficacy estimates ranging from 66.7% to 82.6% depending on the end point evaluated [5–7], leading to regulatory approval and recommendations for adults aged ≥60 years. A third vaccine, the mRNA-based mRNA-1345 (mResvia; Moderna), was approved in 2024. These approvals were followed by the implementation of national vaccination programmes, with recommendations from advisory bodies including the Advisory Committee on Immunization Practices (ACIP) [8] in the United States and the Joint Committee on Vaccination and Immunization (JCVI) [9] in the United Kingdom.

Patients with systemic autoimmune rheumatic diseases (SARDs) represent a population of interest for RSV vaccination. These patients face a dual vulnerability: the immune dysregulation inherent to their underlying disease and the additional immunosuppression conferred by disease-modifying antirheumatic drugs (DMARDs), including conventional synthetic agents such as methotrexate, biologic therapies, and targeted synthetic DMARDs including Janus kinase inhibitors. Population-based data suggest that adults with autoimmune diseases have substantially elevated RSV hospitalization rates compared with the general population [10]; however, all the major RSV vaccine trials excluded patients receiving immunomodulatory therapies, creating a significant evidence gap.

Current vaccination guidelines for patients with rheumatic diseases, including those from the American College of Rheumatology (ACR) and the European Alliance of Associations for Rheumatology (EULAR), do not address RSV vaccination, as both pre-date vaccine availability [11, 12]. Rheumatologists are left without disease-specific guidance on RSV vaccine efficacy, safety, immunogenicity or optimal timing relative to DMARD therapy. This uncertainty is compounded by emerging safety signals—notably an approximately doubled short-term risk of Guillain–Barré syndrome (GBS) following RSV vaccination [13], and by questions regarding the durability of vaccine-induced immunity in immunosuppressed populations.

This review aims to synthesize the available evidence on RSV vaccination in the context of SARDs and DMARD therapy. Our aim is to delineate what is known, identify areas of uncertainty, and highlight knowledge gaps to inform clinical decision-making and future research.

## Methods

### Search strategy

A literature search was conducted using OVID to search MEDLINE and Embase from database inception to 28 January 2026. The primary search strategy was designed to identify studies relating to RSV vaccination or outcomes in patients with SARDs and patients receiving DMARDs. Further details may be found in Supplementary Data S1. No language or date restrictions were applied.

### Supplementary searching

During the review process we identified that several large vaccine effectiveness studies conducted in general populations included subgroup analyses of immunocompromised groups but were not captured by the primary disease-specific search strategy, as their titles, abstracts and indexing terms did not include rheumatic disease or DMARD terminology. Therefore, an additional, supplementary search was conducted to identify relevant, general population RSV vaccination effectiveness studies. This comprised handsearching of high-impact journals (*New England Journal of Medicine*, *The Lancet*, *JAMA*, *JAMA Network Open*, *The Lancet Infectious Diseases*) for RSV vaccine effectiveness studies published until January 2026, with manual review of forest plots and supplementary materials for immunocompromised subgroup data. Backward citation tracking was also performed.

### Screening and eligibility

Titles and abstracts were screened by three reviewers (J.G., V.A. and M.A.). Studies were included if they reported data on RSV vaccination in adults with autoimmune or immunocompromised conditions, RSV disease burden in these populations, or RSV vaccine safety or immunogenicity relevant to immunosuppressed patients. Studies were excluded if they addressed only paediatric populations, were not related to RSV or were duplicates. Full texts of potentially eligible studies were assessed against the inclusion criteria. Studies identified through supplementary searching were included if they reported vaccine effectiveness or safety data stratified by immunocompromised status.

### Data extraction and synthesis

Data were extracted into a standardized template capturing study identification (first author, year, journal, design and country), study design and setting, population size and characteristics (including age, proportion with SARD and DMARDs), the intervention and comparator (including vaccine type and comparator), and outcomes. Extracted outcomes included efficacy or effectiveness estimates, immunogenicity measures (including geometric mean titres and seroconversion rates), safety data (including serious adverse events, GBS, potentially immune-mediated diseases and autoimmune adverse events of special interest), disease flare data, DMARD-specific findings where reported, follow-up duration and study limitations. Each study was also rated for relevance to SARD populations (high, medium, low–medium or low) based on whether it reported SARD-specific outcomes, immunocompromised subgroup data or general population data only.

Given the anticipated study heterogeneity, quantitative meta-analysis was not planned. Findings are synthesized narratively, organized by theme: RSV disease burden in SARD populations, vaccine effectiveness in immunocompromised patients, immunogenicity data, safety profile including the GBS signal, autoimmune adverse events and DMARD-specific considerations.

### Use of artificial intelligence

Claude.ai (Anthropic, Claude Opus) was used for editorial assistance, construction of the data extraction spreadsheet and Python coding for figure generation. All outputs were reviewed and verified by the authors. Claude.ai was not involved in study design, literature searching, screening, interpretation of findings or formulation of conclusions. The authors conducted all substantive scientific work and retain full responsibility.

## Results

### Search results and study characteristics

The primary SARD- and DMARD-specific literature search identified 300 records. After removal of 51 duplicates, 249 unique records were screened. Of these, 206 were excluded (not RSV-related or paediatric population only), leaving 43 studies identified in the primary search for inclusion. The supplementary search of high-impact journals to identify general population vaccine effectiveness studies reporting relevant immunocompromised subgroup data identified a further six studies not captured by the primary search strategy. In total, 49 studies were included in the narrative synthesis (Fig. 1).

**Figure 1:**
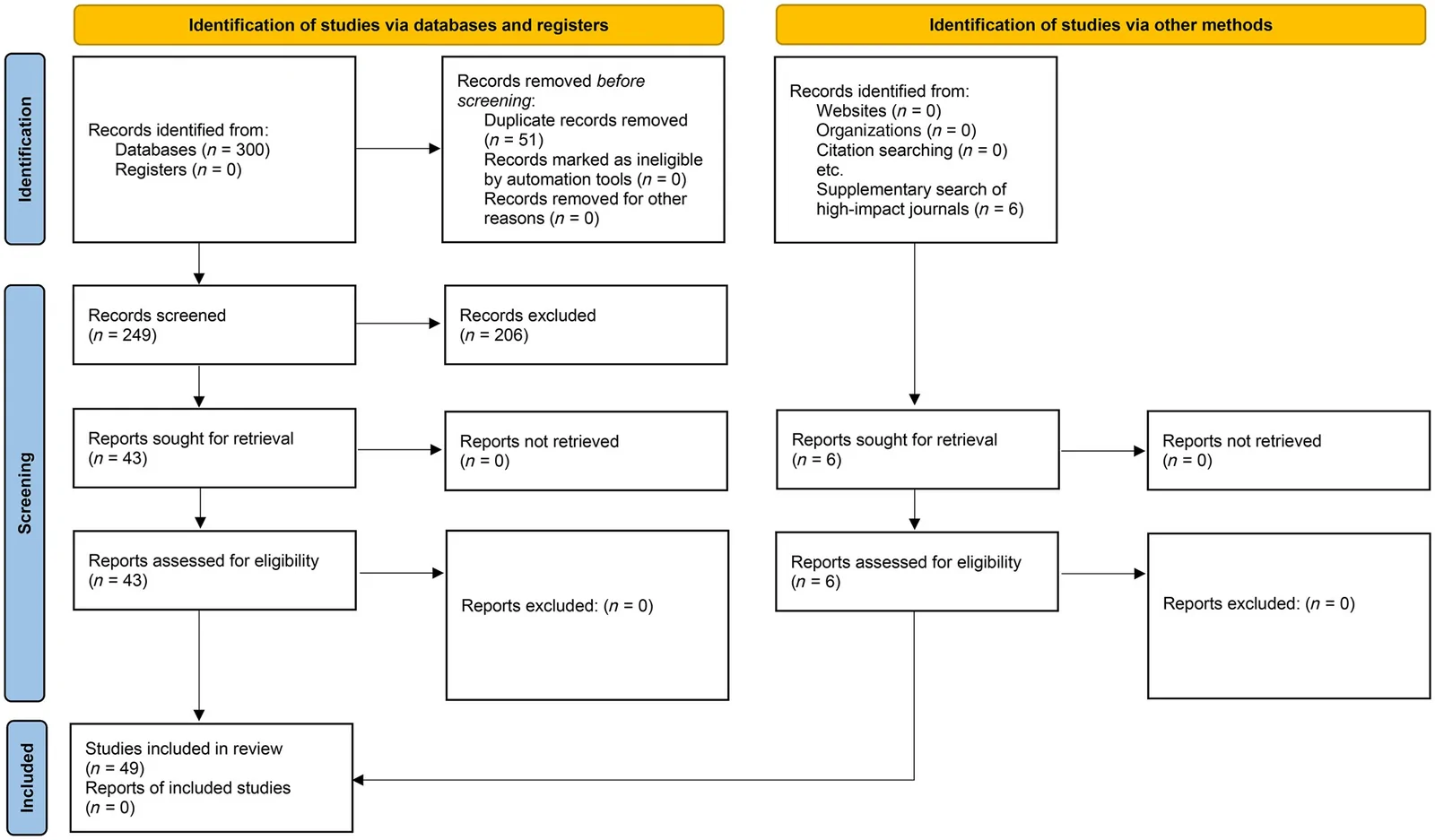
PRISMA 2020 flow diagram. Flow diagram showing the identification, screening, and inclusion of studies. The primary database search identified 300 records. After removal of 51 duplicates, 249 records underwent title and abstract screening, of which 206 were excluded. Supplementary handsearching of high-impact journals identified six additional studies containing immunocompromised subgroup data. A total of 49 studies were included in the narrative synthesis. Source: Page MJ, *et al*. BMJ 2021;372:n71. doi: 10.1136/bmj.n71. This work is licensed under CC BY 4.0. To view a copy of this licence, visit https://creativecommons.org/licenses/by/4.0/

Of these 49 studies, 16 were rated as high relevance to SARD populations, 18 as medium, 2 as low-medium, and 13 as low relevance (Supplementary Table S1). Study designs comprised randomized controlled trials (*n* = 10), retrospective cohort and case–control studies (*n* = 15), pharmacovigilance and post-marketing surveillance analyses (*n* = 8), systematic reviews and meta-analyses (*n* = 2), case reports (*n* = 4), and other designs including editorials, guidelines and commentaries (*n* = 10). The characteristics of included studies are summarized in Table 1.

**Table 1:** Characteristics of included studies (*n* = 49).

Characteristic	*n* (%)
Study design	
Randomized controlled trial	10 (20)
Observational (cohort/case–control)	15 (31)
Pharmacovigilance/post-marketing surveillance	8 (16)
Systematic review/meta-analysis	2 (4)
Case report/series	4 (10)
Other (editorial, guideline, commentary, narrative review)	10 (20)
Relevance to SARD populations	
High	16 (33)
Medium	18 (37)
Low–medium	2 (4)
Low	13 (27)
Geographic setting	
USA	23 (47)
International/multinational	15 (31)
Europe	6 (12)
Canada	2 (4)
Other	3 (6)
Identification source	
Primary database search (OVID MEDLINE/Embase)	43 (88)
Supplementary handsearching	6 (12)

Percentages may not sum to 100% due to rounding. Study design categories are mutually exclusive. SARD: systemic autoimmune rheumatic disease.

### RSV disease burden and vaccination uptake in SARD populations

#### Indirect evidence from immunosuppressed populations

Population-based data from Ontario reported RSV hospitalization rates of 43.6 per 100 000 in adults with autoimmune disorders, compared with 16.2 per 100 000 in the overall adult population and 1.2 per 100 000 in adults with no comorbidities [10]. After adjustment for age and comorbidities, autoimmune disease was associated with a modest independent increase in RSV-related hospitalization risk [adjusted odds ratio (aOR), 1.18]. The crude rate in adults with immunocompromising conditions was higher still (103.9 per 100 000), though this category is dominated by haematological malignancy and solid organ transplant recipients. Hospitalization rates showed a steep age gradient, rising from 2.0 per 100 000 in adults aged 18–49 to 133.5 per 100 000 in those aged ≥80. Only 25% of RSV-associated hospitalizations had RSV coded as the primary diagnosis; the majority were admitted primarily for chronic obstructive pulmonary disease exacerbations, heart failure decompensation or other conditions with RSV detected concurrently. Thirty-day mortality was 10.3% and intensive care unit admission 19.3%.

#### SARD-specific evidence

A retrospective cohort study of SARD patients at Mass General Brigham (*n* = 188) reported that 51% of those with documented RSV infection required hospitalization, 10% required intensive care and 4% died [14]. Frailty was the strongest predictor of adverse outcomes [odds ratio (OR) 3.84]. Among a subgroup with interstitial lung disease, 8 of 23 patients (35%) died.

In recent years the RSV vaccine landscape has changed rapidly (Fig. 2) with multiple factors affecting vaccine uptake. A retrospective cohort study of 10 587 SARD patients aged ≥60 years eligible for RSV vaccination within the Mass General Brigham health system identified 1075 (10.2%) who received RSV vaccine between May 2023 and February 2025 [15]. Factors independently associated with higher odds of vaccination included interstitial lung disease (aOR 1.53; 95% CI: 1.14, 2.04), prior influenza and COVID-19 vaccination, and higher neighbourhood income. Notably, glucocorticoid use was independently associated with substantially lower vaccination odds (aOR 0.33; 95% CI: 0.23, 0.49), representing a paradox whereby patients at highest risk of severe RSV disease were least likely to be vaccinated. Patients identifying as black had lower vaccination odds (aOR 0.64; 95% CI: 0.42, 0.98). No association was observed between specific SARD diagnosis or DMARD class and vaccination uptake.

**Figure 2:**
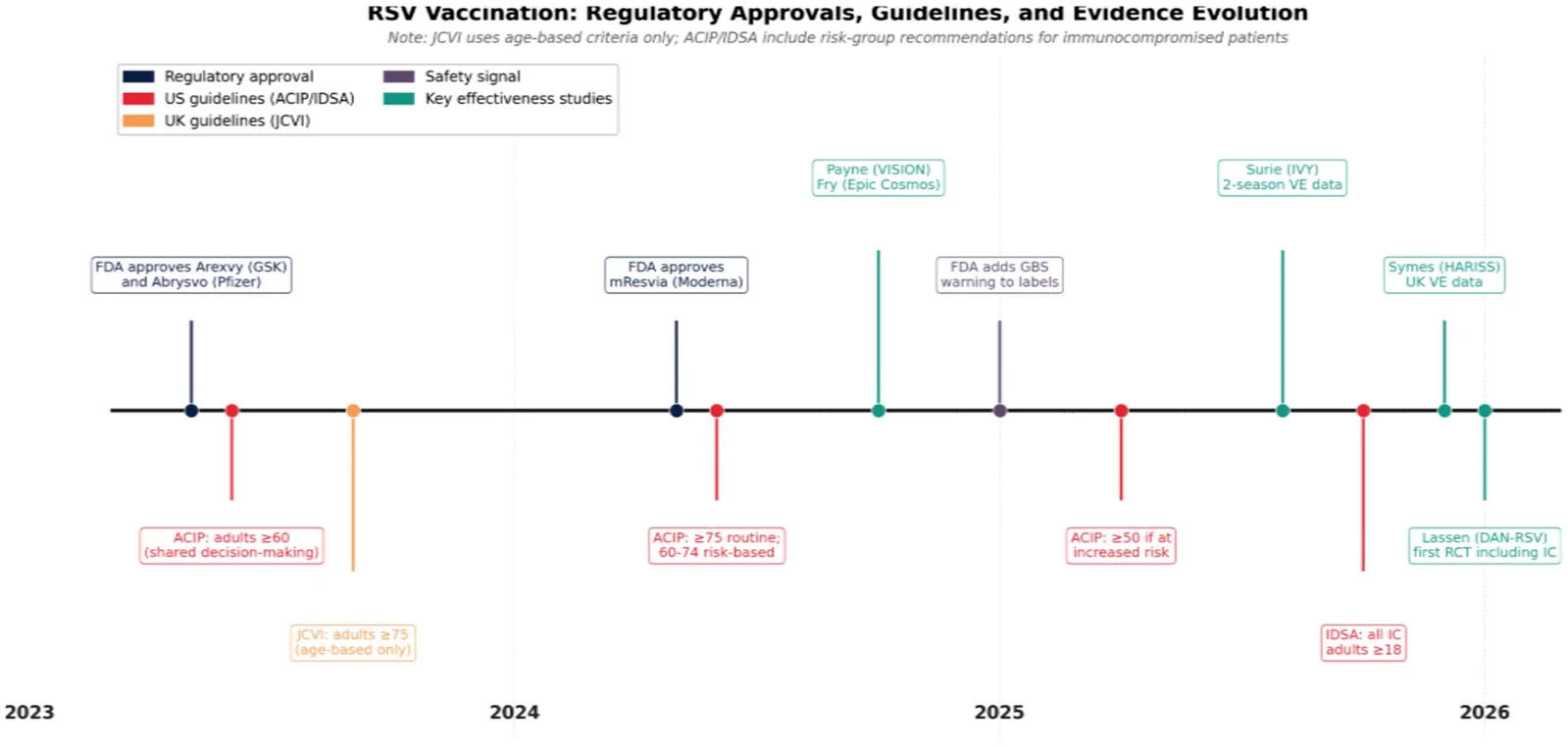
Timeline of respiratory syncytial virus (RSV) vaccine regulatory approvals, guidelines and key evidence. Timeline showing the evolution of RSV vaccination policy from May 2023 to January 2026. Regulatory approvals (dark blue), US guidelines from ACIP and IDSA (red), UK guidelines from JCVI (orange), safety signals (purple) and key effectiveness studies (teal) are indicated. Note the divergence between JCVI (age-based criteria only, from age 75) and ACIP/IDSA (risk-based recommendations including immunocompromised patients from ages 50 and 18, respectively)

### Vaccine effectiveness

#### Indirect evidence

##### Evidence from general population studies

The DAN-RSV pragmatic trial in Denmark (*n* = 131 276) reported vaccine effectiveness (VE) of 83.3% (95% CI: 42.9, 96.9) against RSV-related hospitalization [16]. DAN-RSV is the first randomized trial without formal immunosuppression exclusion criteria; 2.3% of participants had rheumatic disease and 4.0% had immunodeficiency, although subgroup estimates were imprecise (VE in immunosuppressed: 60.5%; 95% CI: −147.3, 96.1). Real-world effectiveness studies from the United States and England reported estimates of 75–83% against hospitalization in unselected older adult populations [17–19].

#### Evidence from studies in immunocompromised populations

The comparative vaccine effectiveness between immunocompetent and immunocompromised patients is described in Table 2 and Fig. 3. Four studies reported vaccine effectiveness stratified by immunocompromised status. The VISION network study (*n* = 36 706 hospitalizations) reported effectiveness of 73% (95% CI: 48, 85) in immunocompromised patients compared with 80% (95% CI: 71, 85) in immunocompetent patients [18]. Rheumatological and inflammatory conditions accounted for 6% of the cohort (*n* = 2347), although a separate vaccine effectiveness estimate for this subgroup was not reported. Data from Epic Cosmos (a de-identified electronic health record database containing routinely collected data from participating healthcare organizations across the United States; *n* = 787 822 tested) showed effectiveness of 67–73% in immunocompromised adults depending on age group, compared with 75% overall [17]. The English HARISS surveillance network reported effectiveness of 72.8% (95% CI: 39.5, 89.3) in immunosuppressed patients (27% of the cohort, defined per the UK Green Book) *vs* 86.2% (95% CI: 73.6, 93.6) in immunocompetent patients [19].

**Table 2:** Vaccine effectiveness against respiratory syncytial virus (RSV)-associated hospitalization in immunocompromised populations.

Study	Design	*n*	IC definition	VE immunocompromised (95% CI)	VE immunocompetent (95% CI)
First-season vaccine effectiveness					
Lassen 2026 (DAN-RSV) [16]	RCT	131 276	Registry-defined immunosuppression	60.5% (−147.3, 96.1)^a^	92.3% (48.8, 99.8)
Payne 2024 (VISION) [18]	Test-negative	36 706	Immunocompromised (ICD-10 diagnosis codes)	73% (48, 85)	80% (71, 85)
Fry 2025 (Epic Cosmos) [17]	Test-negative^b^	787 822	Immunocompromised diagnosis codes	67, 73%^c^	75%^d^
Symes 2026 (HARISS) [19]	Test-negative	1006	UK Green Book definition	72.8% (39.5, 89.3)	86.2% (73.6, 93.6)
Waning effectiveness (two-season data)					
Surie 2025 (IVY Network) [20]	Test-negative	6958	Moderate/severe immunocompromise (COVID-eligibility-adapted definition)		
Same-season				54% (−2, 79)	74% (53, 85)
Prior-season				11% (−51, 48)	62% (40, 76)
Severely immunocompromised populations					
Fry 2025 (HSCT recipients) [17]	Test-negative^b^	—	Haematopoietic stem cell transplant	29, 44%^c^	75%^d^

a Only seven events in the immunosuppressed subgroup; the estimate is uninformative and its confidence interval overlaps the immunocompetent estimate.

b Test-negative case–control design for effectiveness (self-controlled case series for the safety analysis).

c Range across age groups.

d Comparator is the overall cohort (medically attended acute respiratory illness; effectiveness against hospitalization was near-identical). Same-season *vs* prior-season (Surie 2025): same-season = RSV vaccine received during the RSV season in which effectiveness was assessed (median ≈ 3 months before admission); prior-season = vaccine received during the preceding RSV season (median ≈ 14 months before admission). Categories are defined by calendar windows keyed to the hospitalization season (season start 1 August), not by a fixed interval since vaccination. HSCT: haematopoietic stem cell transplant; IC: immunocompromised; RCT: randomized controlled trial; VE: vaccine effectiveness.

**Figure 3:**
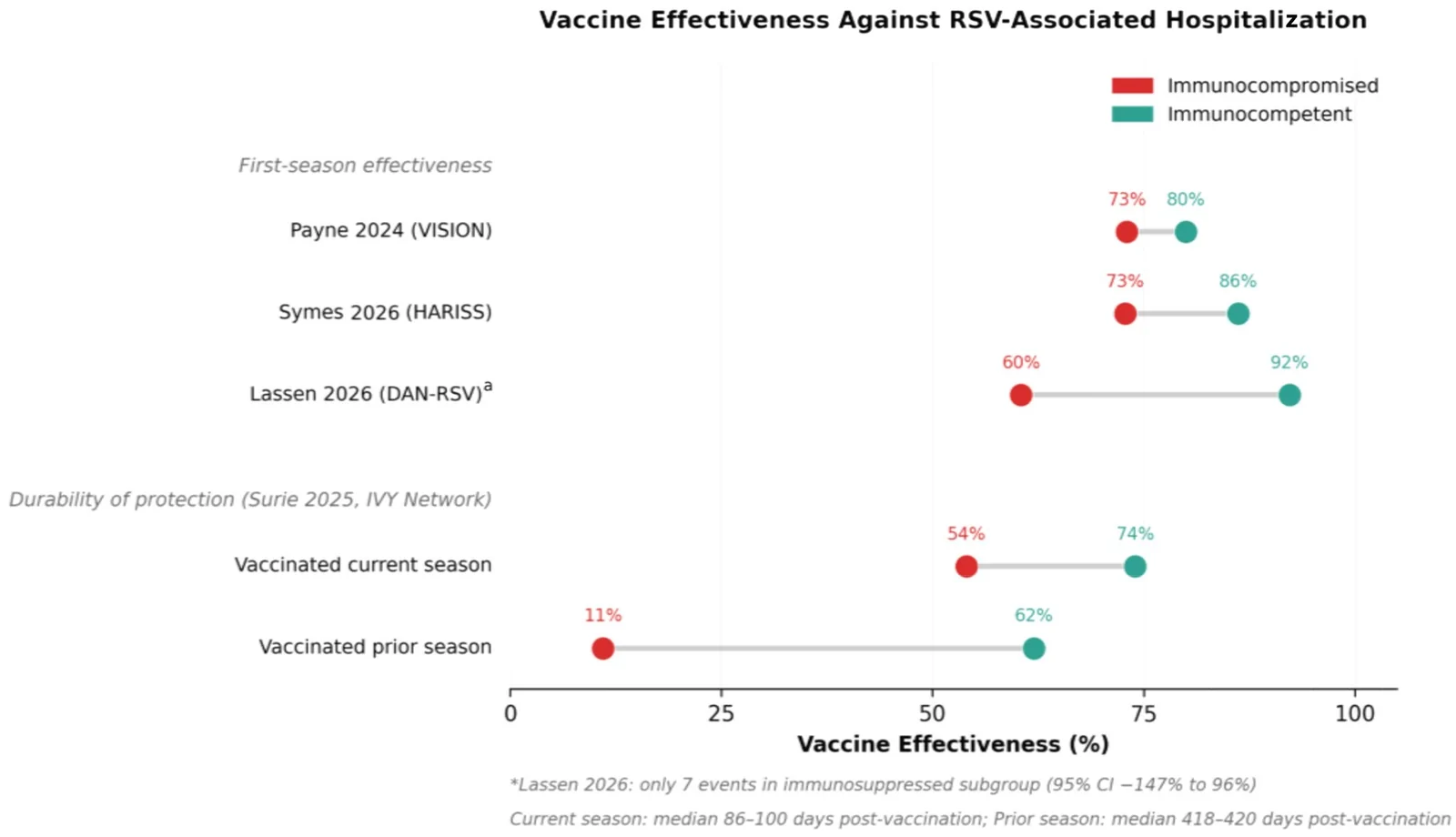
Vaccine effectiveness against respiratory syncytial virus (RSV)-associated hospitalization in immunocompromised *vs* immunocompetent populations. Dumbbell chart showing point estimates of vaccine effectiveness (%) for immunocompromised (red) and immunocompetent (teal) adults from four observational studies. The upper panel shows first-season effectiveness; the lower panel shows durability data from the IVY Network (Surie 2025 [20]), comparing effectiveness in those vaccinated in the current season (median 86–100 days post-vaccination) versus the prior season (median 418–420 days post-vaccination). Horizontal bars connect paired estimates to illustrate the attenuation gap. ^a^Lassen 2026 [16] (DAN-RSV): only seven events in immunosuppressed subgroup; 95% CI: −147%, 96%

#### SARD-specific evidence

In the cohort study of SARD patients aged ≥60 years at Mass General Brigham, among the 1075 vaccinated patients, nine (0.8%) experienced breakthrough RSV infection, all aged >65 years and all occurring >90 days post-vaccination; two required hospitalization and none died [15].

### Waning effectiveness and durability

#### Indirect evidence from studies in immunocompromised populations

The CDC IVY Network assessed vaccine effectiveness over two RSV seasons (*n* = 6958) [20]. Effectiveness in immunocompromised patients was significantly lower than in immunocompetent patients [30% (95% CI: −9, 55) *vs* 67% (95% CI: 53, 77); *P *= 0.02]. Among immunocompromised patients, effectiveness was 54% (95% CI: −2, 79) when vaccination occurred in the same season, declining to 11% (95% CI: −51, 48) when vaccination had occurred in the prior season. In contrast, among immunocompetent patients, effectiveness remained 62% (95% CI: 40, 76) following prior-season vaccination. Use of immunosuppressive therapy was significantly more common among vaccinated breakthrough cases than among unvaccinated cases (33.3% *vs* 16.1% of unvaccinated cases, *P *< 0.001). Another study found that haematopoietic stem cell transplant recipients had markedly reduced vaccine effectiveness of 29–44% depending on age [17].

#### SARD-specific evidence

No studies reported vaccine effectiveness stratified by SARD diagnosis or DMARD class.

### Immunogenicity

#### Indirect and extrapolated evidence from immunosuppressed patients

A prospective cohort study of 38 immunocompromised adults (82% solid organ transplant recipients, 13% with autoimmune or inflammatory conditions) found that only 61% achieved seroconversion following RSV vaccination, with 58% reaching high-titre neutralization thresholds; adjuvanted vaccine (Arexvy) was significantly superior to non-adjuvanted vaccine (Abrysvo) for high-titre responses (79% *vs* 21%, *P *= 0.02) [21]. Evidence extrapolated from other vaccine platforms is broadly consistent with these estimates. Vicente-Rabaneda *et al.* estimated that ∼40% of immunocompromised patients fail to achieve adequate seroconversion thresholds, extrapolating from influenza and COVID-19 vaccine data [22].

#### SARD-specific evidence

No studies reported RSV vaccine immunogenicity data specifically in SARD patients. The MONeT trial, a phase 3 trial of mResvia (mRNA-1345), enrolled 2654 immunocompromised adults including those with autoimmune and inflammatory conditions requiring chronic immunosuppression [23]. The safety profile was reported as comparable to immunocompetent trials. Immunogenicity and efficacy data are awaited.

### RSV vaccination safety

Table 3 summarises the safety of the RSV vaccine with relevance to SARD populations.

**Table 3:** Safety findings relevant to SARD populations.

Category	Source	Key finding	SARD considerations
Guillain–Barré syndrome			
GBS incidence	Stowe 2025 (England) [28]	23 excess cases per million doses (Abrysvo); relative incidence 3.34 (95% CI: 2.12, 5.28); cases cluster on days 8–21 (0–42 day risk window)	Unknown whether immunosuppression modifies risk
	Lloyd 2025 (US Medicare) [29]	Arexvy: IRR 2.46 (1.19, 5.08), *P* = 0.01; Abrysvo: IRR 2.02 (0.93, 4.40), NS	No immunocompromised subgroup reported
	Fry 2025 (Epic Cosmos) [17]	Abrysvo excess ∼18/million; Arexvy excess ∼5/million	—
Risk–benefit	Stowe 2025 [28]	∼1128 RSV hospitalizations and 86 deaths prevented per million doses *vs* ∼23 GBS cases per million (∼49 hospitalizations prevented per GBS case)	Balance may differ in younger SARD patients
Autoimmune adverse events			
RA/autoimmune AEs	Donahue 2025 (VSD) [26]	*n* = 436 823 vaccinated; an ITP signal was detected (aRR 3.04) but not confirmed on medical-record review (most cases pre-dated vaccination); no confirmed autoimmune signal	Reassuring for SARD patients
	Cullen 2025 (Scotland) [27]	Observed-to-expected RA/polyarthritis hospitalizations lower than expected (O/E ratio 0.58; 95% CI: 0.34, 0.93); comprised all RA/polyarthritis hospitalizations, not restricted to new-onset diagnoses	No excess autoimmune risk detected
Case reports	El Labban 2024 [24]	PMR onset day 3 post-Abrysvo; responded to prednisone	Temporal association; causation uncertain
	Seby 2025 [32]	Thrombotic microangiopathy <24 h post-vaccine (Arexvy); complement-mediated mechanism proposed but not confirmed	Mechanism unconfirmed; relevance to complement-driven SARDs uncertain
Disease flare (other vaccines)	Li 2024 (meta-analysis) [42]	SLE flare risk after influenza/COVID-19 vaccines: OR 1.00 (95% CI: 0.14, 7.28; based on a single study)	Extrapolated to RSV; direct data awaited
Potentially immune-mediated diseases			
Phase 3 trial data	Ferguson 2024/2025 [30, 31]	Arexvy: 8 pIMDs within 6 months, 1 vaccine-related (cold-type haemolytic anaemia); balanced *vs* placebo (12-month follow-up)	IC patients excluded from pivotal trials
Immunocompromised	Almeida 2025 (MONeT) [23]	Safety profile comparable to immunocompetent trials; autoimmune adverse events not separately reported	First safety data in an IC population (single-arm)
Safety in immunocompromised populations			
Overall profile	Almeida 2025 (MONeT) [23]	AE profile similar to immunocompetent trials in IC adults; cohort included patients on immunomodulatory therapy (47.8%, mostly DMARDs)	Efficacy data pending

AE: adverse event; aHUS: atypical haemolytic uraemic syndrome; APS: antiphospholipid syndrome; GBS: Guillain–Barré syndrome; IC: immunocompromised; IRR: incidence rate ratio; ITP: immune thrombocytopenia; NS: not significant; O/E: observed-to-expected; pIMD: potentially immune-mediated disease; PMR: polymyalgia rheumatica; RA: rheumatoid arthritis; RSV: respiratory syncytial virus; SARD: systemic autoimmune rheumatic disease; SLE: systemic lupus erythematosus; VSD: Vaccine Safety Datalink.

#### Indirect evidence from general population studies

##### Autoimmune adverse events

Case reports have described new-onset autoimmune conditions following RSV vaccination, including polymyalgia rheumatica with onset 3 days post-Abrysvo vaccination and autoimmune myopathy temporally associated with RSV infection in a vaccinated patient [24, 25].

The Vaccine Safety Datalink study (*n* = 436 823 RSV-vaccinated adults) found no confirmed signals for immune thrombocytopenia or other autoimmune conditions of interest [26]. Observed rates of rheumatoid arthritis and polyarthritis hospitalizations following RSV vaccination were lower than expected (observed-to-expected ratio 0.58) [27].

Evidence suggests a small increased risk of GBS following RSV vaccination, with estimates ranging from ∼5 to 23 excess cases per million doses (relative risk estimates between 2.0 and 3.3) with risk concentrated around 8–21 days after vaccination [17, 28, 29]. Risk–benefit analyses estimate that ∼50 RSV hospitalizations may be prevented per excess GBS case in the target age group [28].

#### Potentially immune-mediated diseases

Potentially immune-mediated disease (pMID) is a term used in vaccine safety literature to describe adverse events or clinical syndromes for which an immune-mediated mechanism is plausible but not confirmed. The significance of reported events in this category should be interpreted with caution as temporal association with vaccination alone does not establish causality. Phase 3 trial data reported pMID rates that were balanced between vaccine and placebo groups. Extended follow-up of Arexvy recipients documented eight pIMD cases within 6 months, of which one (cold-type haemolytic anaemia) was assessed as vaccine-related [30, 31]. A single case of thrombotic microangiopathy was reported following RSV vaccination [32]. The MONeT trial in immunocompromised patients reported a safety profile comparable to immunocompetent trials; detailed pIMD data are awaited [23].

#### SARD-specific evidence

There are no studies evaluating GBS risk, autoimmune adverse events or PMIDs post-RSV vaccination in SARD patients specifically.

### DMARD-specific considerations

No studies reported RSV vaccine efficacy, effectiveness, or immunogenicity stratified by DMARD class or individual drug. All major RSV vaccine trials excluded patients receiving immunomodulatory therapy.

#### Indirect evidence from other vaccine platforms

Evidence for DMARD-specific considerations is lacking and must be extrapolated from other vaccine platforms including influenza and COVID-19 vaccine data. Temporary interruption of MTX has the strongest evidence base for improving vaccine immunogenicity [33, 34]. Rituximab [35] and JAK inhibitors [36] are the therapies most consistently associated with impaired vaccine responses. In contrast, most other biologics appear to have comparatively modest effects.

Vicente-Rabaneda *et al.*, drawing on such influenza vaccine data, recommended considering a 2-week methotrexate hold following RSV vaccination to optimize immunogenicity [22]. Urquiaga *et al.* recommended that rituximab-treated patients receive RSV vaccination timed to coincide with the next scheduled infusion, with rituximab delayed by at least 2 weeks post-vaccination where clinically feasible [37]. As discussed above, both sets of authors noted that these recommendations are based on extrapolation from other vaccine platforms, rather than RSV-specific evidence. A summary of the evidence base for RSV vaccination and associated strategies can be found in Table 4.

**Table 4:** Evidence base for RSV vaccination and associated strategies in patients with SARDs.

Topic	Evidence-base classification	Summary of findings	Applicability to SARD patients
RSV disease burden	Direct/indirect^a^	Limited SARD^b^-specific data. Immunosuppressed patients appear to be at greater risk. Age and frailty are major predictors of adverse outcomes	Relevant, but improved SARD-specific estimates are needed
RSV vaccine effectiveness	Indirect	RSV vaccination reduces RSV-associated lower respiratory tract disease and hospitalization in older adults. Effectiveness may be lower in immunocompromised groups. Breakthrough infections have been reported in SARD patients	Supportive, but definitive estimates of vaccine effectiveness in SARD patients are lacking. Data from the MONeT trial awaited
Waning effectiveness and durability	Indirect	Evidence of waning effectiveness in immunocompromised groups compared with immunocompetent populations. No SARD-specific evidence	Useful context, but SARD-specific studies are required
Repeat dosing	Extrapolated/none	No studies evaluating SARD-specific RSV vaccination strategies	Important evidence gap. Insurance coverage and vaccine availability in publicly funded programmes may be relevant
Immunogenicity	Indirect/extrapolated	Evidence of reduced seroconversion following RSV vaccination in immunosuppressed groups. Data from other vaccine platforms demonstrate similar findings. No studies reporting RSV vaccine immunogenicity data in SARD patients	Useful context, but no direct evidence. Data from the MONeT trial awaited
RSV vaccine safety	Indirect	General population data suggest a small increase in GBS risk following RSV vaccination. Isolated case reports of other new-onset autoimmune diseases and pIMDs. The MONeT trial in immunocompromised patients reported a safety profile comparable to immunocompetent trials	Broadly reassuring, but SARD-specific data remain lacking
DMARD-specific considerations	Extrapolated	No evidence for the impact of DMARDs on RSV vaccination specifically. Evidence from other vaccine platforms suggests JAK inhibitors and rituximab are most consistently associated with impaired vaccine responses	Useful context, but RSV vaccination studies are required

Evidence-base classification: Direct: data derived specifically from SARD populations receiving RSV vaccination; Indirect: data from RSV vaccination in general or other immunocompromised populations; Extrapolated: data from other vaccine platforms (influenza and COVID-19 vaccines).

DMARD: disease-modifying antirheumatic drug; GBS: Guillain–Barré syndrome; IC: immunocompromised; JAK: Janus kinase; pIMD: potentially immune-mediated disease; PMR: polymyalgia rheumatica; RSV: respiratory syncytial virus; SARD: systemic autoimmune rheumatic disease.

a Consider, if feasible to do so, reporting the number of records identified from each database or register searched (rather than the total number across all databases/registers).

b If automation tools were used, indicate how many records were excluded by a human and how many were excluded by automation tools.

## Discussion

This review synthesized 49 studies to address the rapidly evolving literature on RSV vaccination in SARDs, an area where direct evidence remains sparse. Only one study reported RSV vaccine outcomes in SARD patients specifically [14], and one unpublished trial has enrolled immunocompromised patients, including those receiving immunomodulatory therapy [23]. Most available evidence derives from general population vaccine effectiveness studies, pharmacovigilance studies or extrapolation from other vaccine platforms. Despite these limitations, several relevant conclusions may be drawn and evidence gaps identified. These may be used to support clinical decision-making and to guide future research priorities rather than to establish definitive estimates of SARD-specific RSV vaccine effectiveness or safety.

One question is the extent to which RSV poses a genuine risk to SARD patients. A challenge in addressing this evidence gap is the uncertainty surrounding disease attribution. Many epidemiological studies use ‘RSV-associated hospitalization’ as an outcome. This captures admissions temporally linked to RSV detection, but not necessarily those directly caused by infection. In the Ontario population-based cohort, only 25% of RSV-associated hospitalizations had RSV coded as the most responsible diagnosis [10]. In administrative datasets, RSV may be detected in patients admitted for other conditions, raising questions as to whether RSV represents a primary pathogen, a contributory trigger, or an incidental finding. This distinction is particularly relevant for immunocompromised populations, who may undergo more frequent viral testing and experience prolonged viral shedding. Both factors may inflate apparent RSV detection rates without reflecting true infection-related morbidity. In addition, patients with severe presentations may be more likely to undergo viral testing than people with mild disease, potentially leading to overestimates of the proportion of cases that cause severe disease. These issues suggest that estimates of RSV burden in SARD populations may overstate RSV-attributable risks.

Despite these methodological issues, several factors argue against RSV being merely a bystander pathogen. Across randomized and observational studies, vaccination reduces RSV-associated hospitalization by ∼75–85% [16–19] in general populations, an effect that would be unlikely if most viral detections were unrelated to clinical disease. In one study, patients hospitalized with RSV had a greater odds of mortality within 1 year of admission than patients with influenza, (OR = 1.3; 95% CI: 1.0, 1.6) [38]. Other research found that hospitalized adults aged ≥60 years with RSV had a higher odds of invasive mechanical ventilation or death than hospitalized influenza patients (aOR 2.08; 95% CI: 1.33, 3.26) [39]. Severe outcomes, including intensive care admission and mortality, have been reported in a significant proportion of patients with documented RSV infection [10], including in the single SARD-specific cohort [14]. SARD patients with pulmonary disease appear to be at particularly high risk of severe disease. Together, these observations suggest that RSV is likely to be a clinically relevant pathogen in at-risk individuals, although the precise proportion of hospitalizations that are directly attributable to RSV remains challenging to quantify.

Another important consideration with existing disease-burden estimates is the use of broad disease categories. Studies may group different conditions into ‘autoimmune disease’ or ‘immunocompromised’ groups. These categories encompass diseases with different immunopathologies, treatment regimens and comorbidity profiles. For example, adults who have undergone solid organ or haematopoietic stem cell transplants may have substantially higher risks of severe disease than many patients with SARDs. This heterogeneity may limit the applicability of findings to SARD populations. Age is a major risk factor for severe RSV disease [10], with hospitalization rates increasing sharply in older adults. SARDs span a wide age range, and aggregated ‘autoimmune’ estimates may obscure meaningful differences between younger patients with conditions such as systemic lupus erythematosus and older individuals with rheumatoid arthritis. Even well-designed, prospective studies have been unable to assess the burden of RSV-disease in immunosuppressed populations. The New Zealand Respiratory Syncytial Virus-Associated Hospitalizations study noted insufficient data to investigate immunosuppression as a risk factor [40], underscoring the evidence gaps highlighted in this review. These considerations highlight that while autoimmune conditions appear to be associated with an increased risk of severe RSV, the magnitude of this excess risk attributable specifically to SARDs remains uncertain and is likely to vary by age, comorbidity profiles, the specific condition and treatment exposures.

Whether the RSV vaccine is effective in SARD populations is an important question. Observational studies have demonstrated moderate-to-high first-season effectiveness against RSV-associated hospitalization in immunocompromised adults, although efficacy appears to be attenuated by 10–15% compared with immunocompetent populations [17–20]. This pattern mirrors experience with other vaccines in immunosuppressed populations, in which efficacy is reduced but remains clinically meaningful [41]. Important qualifications apply to these observations. The disease-attribution problem discussed above may affect vaccine effectiveness estimation. If a proportion of ‘RSV-associated hospitalizations’ represent incidental detection, apparent effectiveness will be diluted by events that vaccination could not have prevented. The true effectiveness against RSV-disease may therefore be higher than measured estimates suggest. In addition, ‘immunocompromised’ cohorts in these studies were dominated by transplant recipients and patients with haematological malignancies who may have different vaccine response profiles. SARD patients constituted only a minority. For example, only 6% of the VISION network cohort had rheumatological or inflammatory conditions [18] and no study reported vaccine effectiveness stratified by SARD diagnosis. This absence of disease-specific estimates represents a major evidence gap.

An important finding is the markedly accelerated waning of vaccine effectiveness in immunocompromised individuals, illustrated by the results from the CDC IVY Network [20]. Here, immunosuppressive therapy was significantly over-represented among vaccinated breakthrough cases. Protection appears to decline within 12–18 months, raising questions as to whether the single-dose strategy currently recommended in vaccine guidelines is sufficient for patients receiving immunosuppressive therapy. Although revaccination intervals have not yet been defined, more frequent dosing may be required to maintain protection in high-risk populations. This concern is supported by immunogenicity studies from other vaccine platforms demonstrating reduced seroconversion and antibody titres in immunocompromised patients [42]. Emerging RSV-specific immunogenicity data show similar patterns [17, 20], reinforcing concerns about attenuated and potentially short-lived immune responses in immunosuppressed individuals. Despite these concerns, the question of whether and when repeat doses of RSV vaccine should be administered to patients with SARDs remains unresolved. At present, data to guide recommendations on repeat dosing are limited and the optimal revaccination strategy remains uncertain. Current RSV vaccination guidance in the UK [9] and USA [8] continues to recommend a single dose for eligible adults with no repeat doses advised. Importantly, eligibility for public vaccination programmes and insurance coverage are usually linked to national guidelines, meaning that any additional doses given outside of current guidance may not be routinely available or reimbursed.

The impact of disease-modifying antirheumatic drugs (DMARDs) remains a major evidence gap. All major RSV vaccine trials, RENOIR (Abrysvo), AReSVi-006 (Arexvy) and ConquerRSV (mResvia), excluded patients receiving immunomodulatory therapies, and no study has reported vaccine effectiveness stratified by specific agents. Current recommendations regarding vaccination timing relative to methotrexate or B cell-depleting therapy are extrapolated from influenza and COVID-19 vaccine data [22, 37]. Whether these principles translate to RSV vaccine platforms remains unknown. The ongoing MONeT trial [23], which includes immunocompromised participants, will be critical in addressing this uncertainty. Until such data are available, clinicians must rely on indirect evidence and established vaccination principles for immunosuppressed populations. Overall, available data suggest that RSV vaccination provides clinically meaningful, although attenuated, protection in immunocompromised populations.

The final question addressed in this review is whether the RSV vaccine is safe in SARD patients. Although limited, the data are reassuring. An approximately doubled short-term risk of GBS has been observed; however, this risk is small in absolute terms and substantially outweighed by reductions in RSV-associated hospitalization [28]. Of note, there are no SARD-specific estimates for GBS risk. Large pharmacovigilance datasets have not identified a consistent signal for new-onset autoimmune disease following RSV vaccination. Isolated case reports do not establish causation and should be interpreted with caution and against background incidence. Although a complement-mediated mechanism has been proposed in a single case [32], it was not confirmed, and its relevance to complement-driven conditions remains uncertain. Data from other vaccine platforms provide additional reassurance around SARD flares. A meta-analysis of influenza and COVID-19 vaccines in systemic lupus erythematosus reported no increased flare risk [42]. While immunocompromised patients were excluded from RSV vaccine trials, experience with other vaccines in SARD populations has generally demonstrated acceptable safety profiles, providing indirect reassurance. Nonetheless, ongoing surveillance remains important, particularly for patients receiving intensive immunosuppressive therapy.

These findings have implications for vaccination policy. For SARD patients who meet age-based eligibility criteria, the evidence supports vaccination. Risk appears elevated, first-season vaccine effectiveness is retained, and safety signals are limited. The more complex question concerns younger SARD patients who fall below current age thresholds. International recommendations vary, reflecting genuine uncertainty in the evidence-base. Risk-group-based approaches, such as those adopted in the United States [8], align with the observed increased risk in immunocompromised populations, but direct SARD-specific data are lacking. Purely age-based strategies, such as those currently adopted in the UK [9], may fail to protect younger patients at risk of severe disease due to immunosuppressive therapies or conditions. In this context, shared clinical decision-making that considers individual risk factors, treatment intensity and patient preference represents a pragmatic interim approach.

This review has several strengths. To our knowledge, it is the first to address RSV vaccination specifically in the context of SARDs and DMARD therapy. The search strategy combined disease-specific terms with supplementary handsearching of high-impact journals, increasing the likelihood of capturing data embedded in general population studies. It includes explicit consideration of methodological uncertainties, providing an honest appraisal of the evidence-base. However, important limitations remain. Evidence specific to SARD populations is sparse, and broad ‘immunocompromised’ and autoimmune subgroups are heterogeneous, limiting the precision of any conclusions and potential impact on clinical practice. Effectiveness data for SARD patients therefore remain indirect and no studies report stratification by SARD or DMARD class or agent. When SARD-relevant subgroups have been included in studies, these have been small [21], meaning any signal from these studies is suitable for hypothesis-generation rather than practice-changing recommendations. Many effectiveness estimates are imprecise, reflecting small event numbers and high uncertainty [16]. RSV outcome definitions and ascertainment may vary across studies due to local differences in clinical and laboratory practice. Estimates derived from observational studies may be affected by residual confounding due to differences in frailty, comorbidity burden, healthcare-seeking behaviour, vaccine uptake and immunosuppressive treatment. The rapidly evolving nature of the evidence-base means that conclusions may change as new data emerge.

In summary, RSV poses an important, though incompletely quantified, risk to patients with SARDs. This review should be considered as a summary of the existing evidence, rather than definitive SARD-specific RSV vaccination guidance. Although data are sparse, RSV vaccination appears effective in immunocompromised populations, with an acceptable safety profile but protection may wane more rapidly than in immunocompetent individuals. The absence of DMARD-specific data, uncertainty regarding optimal revaccination intervals and the extent to which RSV disease-burden captured in administrative datasets represents true morbidity are important evidence gaps. Until further evidence is available, specifically from ongoing trials, personalized, shared decision-making remains the most appropriate framework for SARD patients and their treating clinicians.

## Supplementary Material

keag513_Supplementary_Data

## Data Availability

No new data were generated or analysed in support of this article.
