# Supplementary material for: Respiratory syncytial virus vaccination in systemic autoimmune rheumatic diseases: a systematic review
Source: Rheumatology (Oxford). 2026 Sep 17;65(10):keag513. doi: 10.1093/rheumatology/keag513 (PMC13719517; doi:10.1093/rheumatology/keag513)
Supplement: Supplementary file 1 [file keag513_supplementary_data.docx]

Supplementary Data S1.

RSV Review Search Strategy

The search strategy combined four components. The first captured respiratory syncytial virus using subject headings and text word variants (RSV, respiratory syncytial). The second targeted vaccination using vaccine-related subject headings and terms for specific RSV vaccines (Abrysvo, Arexvy, mResvia, RSVpreF, RSVPreF3). The third identified autoimmune rheumatic diseases using subject headings for connective tissue diseases, rheumatic diseases, and autoimmune diseases, supplemented by free-text terms for specific conditions (lupus, rheumatoid arthritis, vasculitis, myositis, scleroderma, Sjögren). The fourth captured disease-modifying therapy using subject headings for immunosuppressive agents, antirheumatic agents, and biological products, alongside free-text terms for specific DMARDs (methotrexate, rituximab, tocilizumab, abatacept, JAK inhibitor, mycophenolate, cyclophosphamide, azathioprine).

Supplementary Table S1.

| **Study** | **Study design** | **Geographic setting** | **Relevance to SARDs** | **Reference** |
| --- | --- | --- | --- | --- |
| **Almeida 2025** | RCT | International/multinational | High | ^1^ |
| **Anastassopoulou 2025** | Pharmacovigilance/post-marketing surveillance | USA | Medium | ^2^ |
| **Anastassopoulou 2025** | Other | International/multinational | High | ^3^ |
| **Anderer 2025** | Other | USA | Low | ^4^ |
| **Awar 2023** | Other | International/multinational | Low | ^5^ |
| **Buchan 2025** | Observational | Canada | High | ^6^ |
| **Buynak 2024** | RCT | International/multinational | Low | ^7^ |
| **Chan 2025** | Other | Other | Low | ^8^ |
| **Cullen 2025** | Observational | Europe | Medium | ^9^ |
| **de Bruin 2025** | Pharmacovigilance/post-marketing surveillance | International/multinational | Low | ^10^ |
| **Donahue 2025** | Pharmacovigilance/post-marketing surveillance | USA | Low | ^11^ |
| **El Labban 2024** | Case report/series | USA | High | ^12^ |
| **Enns 2025** | Observational | USA | High | ^13^ |
| **Ferguson 2024** | RCT | International/multinational | High | ^14^ |
| **Ferguson 2025** | RCT | International/multinational | Medium | ^15^ |
| **Fry 2025** | Observational | USA | High | ^16^ |
| **Fry 2025** | Observational | USA | Medium | ^17^ |
| **Garofalo 2024** | Observational | USA | Low-Medium | ^18^ |
| **Heydari Sirat 2025** | Observational | Other | Low | ^19^ |
| **Ison 2024** | RCT | International/multinational | Medium | ^20^ |
| **Jamshaid 2024** | Case report/series | USA | Medium | ^21^ |
| **Jeong 2025** | Pharmacovigilance/post-marketing surveillance | International/multinational | Low-Medium | ^22^ |
| **Killikelly 2024** | Other | Canada | Low | ^23^ |
| **Kim 2025** | Observational | USA | Medium | ^24^ |
| **Leroux-Roels 2023** | RCT | Europe | Low | ^25^ |
| **Li 2024** | Systematic Review / Meta-analysis | International/multinational | High | ^26^ |
| **Li 2025** | Pharmacovigilance/post-marketing surveillance | USA | Medium | ^27^ |
| **Liang 2025** | Other | USA | Medium | ^28^ |
| **Lloyd 2025** | Observational | USA | High | ^29^ |
| **MacDonald 2025** | Pharmacovigilance/post-marketing surveillance | USA | Medium | ^30^ |
| **Mangan 2025** | RCT | Other | Medium | ^31^ |
| **Mikhail 2024** | Case report/series | USA | Low | ^32^ |
| **Schwarz 2022** | RCT | International/multinational | Low | ^33^ |
| **Scott 2025** | Systematic Review / Meta-analysis | International/multinational | High | ^34^ |
| **Seby 2025** | Case report/series | USA | Medium | ^35^ |
| **Shaaban 2025** | Other | International/multinational | Medium | ^36^ |
| **Simon 2025** | Other | Europe | Low | ^37^ |
| **Stowe 2025** | Observational | Europe | High | ^38^ |
| **Urquiaga 2025** | Other | USA | High | ^39^ |
| **Verheust 2025** | Pharmacovigilance/post-marketing surveillance | International/multinational | Medium | ^40^ |
| **Vicente-Rabaneda 2025** | Other | International/multinational | High | ^41^ |
| **Widagdo 2024** | RCT | USA | Low | ^42^ |
| **Wu 2025** | Pharmacovigilance/post-marketing surveillance | USA | Medium | ^43^ |
| **Karaba 2025** | Observational | USA | High | ^44^ |
| **Davis 2026** | Observational | USA | High | ^45^ |
| **Payne 2024** | Observational | USA | Medium | ^46^ |
| **Surie 2025** | Observational | USA | High | ^47^ |
| **Symes 2025** | Observational | Europe | Medium | ^48^ |
| **Lassen 2026** | RCT | Europe | Medium | ^49^ |

1. Almeida NC, Parameswaran L, DeHaan EN, et al. Immunogenicity and Safety of the Bivalent Respiratory Syncytial Virus Prefusion F Subunit Vaccine in Immunocompromised or Renally Impaired Adults. *Vaccines* 2025;13(3) (no pagination) doi: <https://dx.doi.org/10.3390/vaccines13030328>

2. Anastassopoulou C, Panagiotopoulos AP, Ferous S, et al. RSV vaccines and Guillain-Barre syndrome: Insights into an emerging concern. *Vaccine* 2025;61(no pagination) doi: <https://dx.doi.org/10.1016/j.vaccine.2025.127338>

3. Anastassopoulou C, Medic S, Ferous S, et al. Development, Current Status, and Remaining Challenges for Respiratory Syncytial Virus Vaccines. *Vaccines* 2025;13(2) (no pagination) doi: <https://dx.doi.org/10.3390/vaccines13020097>

4. Anderer S. FDA Issues Warning of Guillain-Barre Syndrome Risk for 2 RSV Vaccines. *Jama* 2025;333(12):1023-24. doi: <https://dx.doi.org/10.1001/jama.2025.0545>

5. Awar M, Mylonakis E. In older adults, an AS01E-adjuvanted RSVPreF3 OA vaccine reduced RSV-related lower respiratory tract disease. *Annals of Internal Medicine* 2023;176(6):JC63. doi: <https://dx.doi.org/10.7326/J23-0039>

6. Buchan SA, Alessandrini J, Drover SSM, et al. Incidence of Respiratory Syncytial Virus-Associated Hospitalization Among Adults in Ontario, Canada, 2017-2019. *Journal of Infectious Diseases* 2025;232(5):e799-e810. doi: <https://dx.doi.org/10.1093/infdis/jiaf343>

7. Buynak R, Cannon K, DeAtkine D, et al. Randomized, Open-Label Phase 3 Study Evaluating Immunogenicity, Safety, and Reactogenicity of RSVPreF3 OA Coadministered with FLU-QIV-HD in Adults Aged >= 65. *Infectious Diseases and Therapy* 2024;13(8):1789-805. doi: <https://dx.doi.org/10.1007/s40121-024-00985-4>

8. Chan JCK, Kwan MYW, Lam W, et al. Importance of surveillance and vaccination in managing respiratory syncytial virus infections among older adults in Hong Kong. *Hong Kong Medical Journal* 2025;31(4):256-61. doi: <https://dx.doi.org/10.12809/hkmj255182>

9. Cullen LA, Gibbons CL, Shi T, et al. RSV Vaccination Programme for Older Adults: A Scotland-Wide Study on RSVpreF Vaccine Safety. *Vaccines* 2025;13(11) (no pagination) doi: <https://dx.doi.org/10.3390/vaccines13111088>

10. de Bruin O, Nab L, Choi J, et al. A Post-Authorisation Safety Study of a Respiratory Syncytial Virus Vaccine in Pregnant Women and Their Offspring in a Real-World Setting: Generic Protocol for a Target Trial Emulation. *Vaccines* 2025;13(3) (no pagination) doi: <https://dx.doi.org/10.3390/vaccines13030272>

11. Donahue JG, Cocoros NM, Kieke BA, et al. Near real-time surveillance and tree-based data mining to assess the safety of respiratory syncytial virus vaccines in older adults in the vaccine safety datalink. *Vaccine* 2025;67(no pagination) doi: <https://dx.doi.org/10.1016/j.vaccine.2025.127873>

12. El Labban M, Oluic S, Guleid H, et al. Polymyalgia Rheumatica Following Respiratory Syncytial Virus (Rsv) Vaccination. *European Journal of Case Reports in Internal Medicine* 2024;11(7) doi: <https://dx.doi.org/10.12890/2024_004636>

13. Enns JP, Wang J, Jiang B, et al. Outcomes of respiratory syncytial virus infection in patients with systemic autoimmune rheumatic disease. *Seminars in Arthritis and Rheumatism* 2025;75(no pagination) doi: <https://dx.doi.org/10.1016/j.semarthrit.2025.152846>

14. Ferguson M, Schwarz TF, Nunez SA, et al. Noninferior Immunogenicity and Consistent Safety of Respiratory Syncytial Virus Prefusion F Protein Vaccine in Adults 50-59 Years Compared to >=60 Years of Age. *Clinical Infectious Diseases* 2024;79(4):1074-84. doi: <https://dx.doi.org/10.1093/cid/ciae364>

15. Ferguson M, Schwarz TF, Nunez SA, et al. Persistence of Immunogenicity and Safety of the Respiratory Syncytial Virus Prefusion F Protein Vaccine (RSVPreF3 OA) Administered to Adults 50-59 Years of Age (YOA). *Open Forum Infectious Diseases* 2025;12(Supplement 1):S522-S23. doi: <https://dx.doi.org/10.1093/ofid/ofae631.907>

16. Fry SE, Terebuh P, Kaelber DC, et al. Effectiveness and Safety of Respiratory Syncytial Virus Vaccine for US Adults Aged 60 Years or Older. *JAMA Network Open* 2025;8(5) (no pagination) doi: <https://dx.doi.org/10.1001/jamanetworkopen.2025.8322>

17. Fry S, Terebuh P, Kaelber D, et al. Real-World Effectiveness and Safety of Respiratory Syncytial Virus Protein Subunit Vaccines in U.S. Adults Aged >=60 Years, 2023-2024. *Journal of General Internal Medicine* 2025;40(Supplement 1):S612. doi: <https://dx.doi.org/10.1007/s11606-025-09516-4>

18. Garofalo D, Lino MM, Mikati T, et al. Background Incidence Rates of Guillain-Barre Syndrome in United States Older Adults at Risk of Respiratory Syncytial Virus in a Trial-Similar Real World Data Cohort. *Pharmacoepidemiology and Drug Safety Conference* 2024;33(Supplement 2) doi: <https://dx.doi.org/10.1002/pds.5891>

19. Heydari Sirat S, Shatizadeh Malekshahi S, Jabbari MR. Prevalence, clinical features, and seasonal patterns of SARS-CoV-2, influenza, and HRSV in patients with a history of kidney transplantation in Iran. *BMC Infectious Diseases* 2025;25(1) (no pagination) doi: <https://dx.doi.org/10.1186/s12879-025-12163-z>

20. Ison MG, Papi A, Athan E, et al. Efficacy and Safety of Respiratory Syncytial Virus (RSV) Prefusion F Protein Vaccine (RSVPreF3 OA) in Older Adults Over 2 RSV Seasons. *Clinical Infectious Diseases* 2024;78(6):1732-44. doi: <https://dx.doi.org/10.1093/cid/ciae010>

21. Jamshaid F, Mallick SA, Alsaadi A, et al. An Unexpected Escalation: A Case of Rsv Myositis. *Chest* 2024;166(4 Supplement):A1083. doi: <https://dx.doi.org/10.1016/j.chest.2024.06.713>

22. Jeong J, Jo H, Park J, et al. Global Estimates of Vaccine-Associated Hepatic Autoimmune Disorders and Their Related Vaccines, 1968-2024: An International Analysis of the WHO Pharmacovigilance Database. *International Archives of Allergy and Immunology* 2025;186(7):696-702. doi: <https://dx.doi.org/10.1159/000542865>

23. Killikelly A, Siu W, Abrams EM, et al. Respiratory syncytial virus vaccination in older adults. *Cmaj* 2024;Canadian Medical Association Journal. 196(29):E1011. doi: <https://dx.doi.org/10.1503/cmaj.240906>

24. Kim HS, Lo NC, Boscardin WJ, et al. Low Uptake and Disparities in Respiratory Syncytial Virus Vaccination Among US Veterans. *Open Forum Infectious Diseases* 2025;12(11) (no pagination) doi: <https://dx.doi.org/10.1093/ofid/ofaf434>

25. Leroux-Roels I, Davis MG, Steenackers K, et al. Safety and Immunogenicity of a Respiratory Syncytial Virus Prefusion F (RSVPreF3) Candidate Vaccine in Older Adults: Phase 1/2 Randomized Clinical Trial. *Journal of Infectious Diseases* 2023;227(6):761-72. doi: <https://dx.doi.org/10.1093/infdis/jiac327>

26. Li XZ, Li YW, Huang CY, et al. Humoral immunity and safety of respiratory virus vaccines in systemic lupus erythematosus population: a meta-analysis based on twenty-five observational studies. *Annals of Medicine* 2024;56(1) (no pagination) doi: <https://dx.doi.org/10.1080/07853890.2024.2392882>

27. Li J, Zhang Z, Wang M. Post-licensure safety of respiratory syncytial virus vaccines, Vaccine Adverse Event Reporting System, United States, May 2023-December 2024. *Preventive Medicine Reports* 2025;56(no pagination) doi: <https://dx.doi.org/10.1016/j.pmedr.2025.103150>

28. Liang C, Chilson EL, Wu J, et al. Can We Compare Attributable Risk of Adverse Events with the Self-Controlled Case Series Design in Vaccine Safety Studies? A Use Case of Guillain-Barre Syndrome. *medRxiv* 2025;08 doi: <https://dx.doi.org/10.1101/2025.05.07.25327155>

29. Lloyd PC, Shah PB, Zhang HT, et al. Evaluation of Guillain-Barre syndrome following Respiratory Syncytial Virus Vaccination among Medicare Beneficiaries 65 Years and Older. *medRxiv* 2025;06 doi: <https://dx.doi.org/10.1101/2024.12.27.24319702>

30. MacDonald SC, Gandhi S, Adimadhyam S, et al. Rapid Surveillance Study to Evaluate Safety of Respiratory Syncytial Virus (RSV) Vaccine (ABRYSVOTM) Exposure During Pregnancy in the United States. *Pharmacoepidemiology and Drug Safety Conference: 41st International Society for Pharmacoepidemiology International Conference, ISPEs* 2025;34(Supplement 1) doi: <https://dx.doi.org/10.1002/pds.70186>

31. Mangan N, Dennis P, Roussy J, et al. Co-Administration of Adjuvanted Respiratory Syncytial Virus Prefusion F Protein Vaccine with Adjuvanted Recombinant Zoster Vaccine in Adults Aged >=50 Years. *Internal Medicine Journal* 2025;55(Supplement 1):67-68. doi: <https://dx.doi.org/10.1111/imj.70003>

32. Mikhail E, Siddiqui MZ, Ghumman A, et al. Acute Demyelinating Polyradiculoneuropathy after the Novel Rsv Vaccination: A Case Report and Literature Review. *Chest* 2024;166(4 Supplement):A2791-A92. doi: <https://dx.doi.org/10.1016/j.chest.2024.06.1691>

33. Schwarz TF, Hwang SJ, Ylisastigui PP, et al. Safety and Reactogenicity of an Investigational Respiratory Syncytial Virus (RSV) Prefusion F Protein Vaccine for Adults >= 60 Years of Age (RSVPreF3 OA): an Interim Analysis at 6 Months after Vaccination. *Open Forum Infectious Diseases* 2022;9(Supplement 2):S108-S09. doi: <https://dx.doi.org/10.1093/ofid/ofac492.197>

34. Scott J, Abers MS, Marwah HK, et al. Updated Evidence for Covid-19, RSV, and Influenza Vaccines for 2025-2026. *New England Journal of Medicine* 2025;393(22):2221-42. doi: <https://dx.doi.org/10.1056/NEJMsa2514268>

35. Seby R, McCormick BJ, Wolf E, et al. Respiratory Syncytial Virus Vaccine Induced Thrombotic Microangiopathy. *Journal of Blood Medicine* 2025;16:331-35. doi: <https://dx.doi.org/10.2147/JBM.S478230>

36. Shaaban FL, Bont LJ. The big five vexing questions of respiratory syncytial virus immunisation. *The Lancet Respiratory Medicine* 2025;13(6):477-78. doi: <https://dx.doi.org/10.1016/S2213-2600%2825%2900093-1>

37. Simon S, Joean O, Welte T, et al. The role of vaccination in COPD: influenza, SARS-CoV-2, pneumococcus, pertussis, RSV and varicella zoster virus. *European Respiratory Review* 2023;32(169) (no pagination) doi: <https://dx.doi.org/10.1183/16000617.0034-2023>

38. Stowe J, Watson CH, Ramsay ME, et al. Assessing the risk of Guillain-Barre syndrome in older adults after bivalent RSV pre-F vaccination in England. *Nature Communications* 2025;16(1) (no pagination) doi: <https://dx.doi.org/10.1038/s41467-025-66280-z>

39. Urquiaga M, Winthrop KL, Curtis JR. Vaccination Update and Specific Concerns for RA. *Current Rheumatology Reports* 2025;27(1) (no pagination) doi: <https://dx.doi.org/10.1007/s11926-025-01197-6>

40. Verheust C, Deraedt Q, Bryan P. Early Post-Marketing Safety Surveillance for the Respiratory Syncytial Virus Prefusion F Protein Vaccine (RSVPreF3 OA). *Open Forum Infectious Diseases* 2025;12(Supplement 1):S113. doi: <https://dx.doi.org/10.1093/ofid/ofae631.175>

41. Vicente-Rabaneda EF, Cardenoso-Domingo L, Castaneda S. Respiratory Syncytial Virus Vaccination. *Jama* 2025;333(22):2021-22. doi: <https://dx.doi.org/10.1001/jama.2025.2137>

42. Widagdo W, Bastian AR, Jastorff AM, et al. Concomitant Administration of Ad26.RSV.preF/RSV preF Protein Vaccine and High-Dose Influenza Vaccine in Adults 65 Years and Older: A Noninferiority Trial. *Journal of Infectious Diseases* 2024;230(2):e374-e83. doi: <https://dx.doi.org/10.1093/infdis/jiad594>

43. Wu J, Bosan R, Huo T, et al. Utilization of Near Real-Time, Pre-Adjudicated Claims Data for Timely Insights on the Risk of Guillain-Barre Syndrome (GBS) Following Respiratory Syncytial Virus (RSV) Vaccines during the Early Post-approval Period. *Pharmacoepidemiology and Drug Safety Conference: 41st International Society for Pharmacoepidemiology International Conference, ISPEs* 2025;34(Supplement 1) doi: <https://dx.doi.org/10.1002/pds.70186>

44. Karaba AH, Hage C, Sengsouk I, et al. Antibody Response to Respiratory Syncytial Virus Vaccination in Immunocompromised Persons. *JAMA* 2025;333(5):429-32. doi: 10.1001/jama.2024.25395

45. Davis NA, Wang J, Wang X, et al. Investigating Factors Associated With Respiratory Syncytial Virus Vaccination and Breakthrough Infection Among Patients With Systemic Autoimmune Rheumatic Diseases. *Journal of Rheumatology* 2026;15:15. doi: <https://dx.doi.org/10.3899/jrheum.2025-0934>

46. Payne AB, Watts JA, Mitchell PK, et al. Respiratory syncytial virus (RSV) vaccine effectiveness against RSV-associated hospitalisations and emergency department encounters among adults aged 60 years and older in the USA, October, 2023, to March, 2024: a test-negative design analysis. *Lancet* 2024;404(10462):1547-59. doi: 10.1016/s0140-6736(24)01738-0

47. Surie D, Self WH, Yuengling KA, et al. RSV Vaccine Effectiveness Against Hospitalization Among US Adults Aged 60 Years or Older During 2 Seasons. *Jama* 2025;334(16):1442-51. doi: 10.1001/jama.2025.15896

48. Symes R, Whitaker HJ, Ahmad S, et al. Vaccine effectiveness of a bivalent respiratory syncytial virus (RSV) pre-F vaccine against RSV-associated hospital admission among adults aged 75&#x2013;79 years in England: a multicentre, test-negative, case&#x2013;control study. *The Lancet Infectious Diseases* 2026;26(3):229-38. doi: 10.1016/S1473-3099(25)00546-8

49. Lassen MCH, Johansen ND, Christensen SH, et al. RSV Prefusion F Vaccine for Prevention of Hospitalization in Older Adults. *N Engl J Med* 2026;394(2):138-51. doi: 10.1056/NEJMoa2509810 [published Online First: 20250830]
